# Biomarkers and genetic determinants of cardiac sarcoidosis: current status, the unmet needs and future perspectives

**DOI:** 10.3389/fcvm.2026.1754375

**Published:** 2026-03-02

**Authors:** Tine Bajec, Matevž Harlander, Nadja Pucelj Koren, Mahwash Kassi, Gregor Poglajen

**Affiliations:** 1 Advanced Heart Failure and Transplantation Center, Department of Cardiology, University Medical Centre Ljubljana, Ljubljana, Slovenia; 2 Department of Pulmonology, University Medical Centre Ljubljana, Ljubljana, Slovenia; 3 Department of Internal Medicine, Faculty of Medicine, University of Ljubljana, Ljubljana, Slovenia; 4 Division of Cardiology, Houston Methodist Hospital, Houston, TX, United States

**Keywords:** biomarker, genetics, heart, sarcoidosis, therapy

## Abstract

Sarcoidosis is a systemic disorder driven by genetic predisposition, environmental exposures, and immune dysregulation, resulting in the formation of noncaseating granulomas across multiple organs. In cardiac sarcoidosis (CS), immune cell infiltration of the myocardium, epicardium, and endocardium may lead to conduction disturbances, ventricular arrhythmias, and heart failure. While overt cardiac involvement was historically considered rare, affecting only 5% of sarcoidosis patients, the wider availability and improved sensitivity of contemporary cardiac imaging have revealed a substantially higher burden, with cardiac involvement reaching up to 55% in selected, systematically screened populations. Current diagnostic approaches for CS, including endomyocardial biopsy (EMB), cardiovascular magnetic resonance (CMR), and fluorine-18 fluorodeoxyglucose–positron emission tomography (FDG-PET), offer valuable insights but are restricted by high costs, invasiveness, and limited sensitivity and specificity. These challenges, together with the disproportionate contribution of cardiac involvement to sarcoidosis-related mortality, underscore the need for innovative, non-invasive, and widely accessible diagnostic strategies. Emerging evidence suggests that novel serum biomarkers and genomic studies hold promise for transforming the diagnostic landscape of CS. Biomarkers may provide accessible, cost-effective tools to complement established diagnostic methods, while genetic insights could identify individuals at higher risk for cardiac involvement and stratify patients based on disease phenotype. This review examines current evidence on serum biomarkers and genetic studies in CS diagnosis, identifies critical knowledge gaps, and proposes future directions aimed at advancing diagnostic precision and improving clinical outcomes.

## Introduction

Sarcoidosis is a systemic inflammatory disorder characterized by noncaseating granulomas that may involve the heart. Cardiac sarcoidosis (CS) occurs either as part of systemic sarcoidosis or as isolated CS, in which granulomatous inflammation is confined to the myocardium (1). Reported cardiac involvement in systemic sarcoidosis ranges from 3.7% to 54.9% and shows substantial ethnic variability (2). Isolated CS may account for approximately 25% of CS cases (1). Given the risk of ventricular arrhythmias, conduction disease, heart failure, and sudden cardiac death, timely and accurate diagnosis remains critical.

Histologic confirmation of myocardial granulomas by endomyocardial biopsy (EMB) is the diagnostic gold standard, but sensitivity is limited by patchy involvement and procedural risks restrict routine use (3). However, the distribution of inflammatory infiltrates limits EMB's sensitivity, while procedural risks often restrict its use to select cases where other diagnostic methods remain inconclusive. Accordingly, major consensus pathways (HRS, JCS, WASOG) support a probabilistic diagnosis integrating clinical features with electrocardiography, echocardiography, cardiovascular magnetic resonance (CMR), and fluorine-18 fluorodeoxyglucose–positron emission tomography (FDG-PET), complemented by extracardiac histology when available (4–6). However, even advanced imaging lacks sufficient specificity to serve as a definitive standalone test, and in practice is used to estimate the likelihood and activity of CS rather than confirm it.

Serum biomarkers offer a readily accessible and non-invasive diagnostic approach, with elevated levels of lysozyme, soluble interleukin-2 receptor (sIL-2R), and angiotensin-converting enzyme (ACE), playing an established role in aiding the diagnosis of systemic sarcoidosis (5). Yet their diagnostic value for isolated CS is limited by low cardiac granuloma burden and by poor specificity for detecting cardiac involvement in systemic disease. In parallel, ethnic variability, familial clustering, and twin concordance support a genetic contribution, but genetic data are not currently incorporated into diagnostic algorithms of sarcoidosis, including CS (7). Advances in data integration and analytic approaches may enable biomarkers and genetic information to improve diagnostic performance while reducing dependence on invasive and costly testing.

Based on a targeted PubMed/MEDLINE search for studies on sarcoidosis/cardiac sarcoidosis biomarkers and genetics, this review summarizes the evidence for serum biomarkers and genetic studies in detecting cardiac involvement in systemic sarcoidosis and in isolated CS, and proposes an evidence-based framework to clarify current clinical utility and key unmet diagnostic needs.

## Markers of granulomatous inflammation

**Figure 1:**
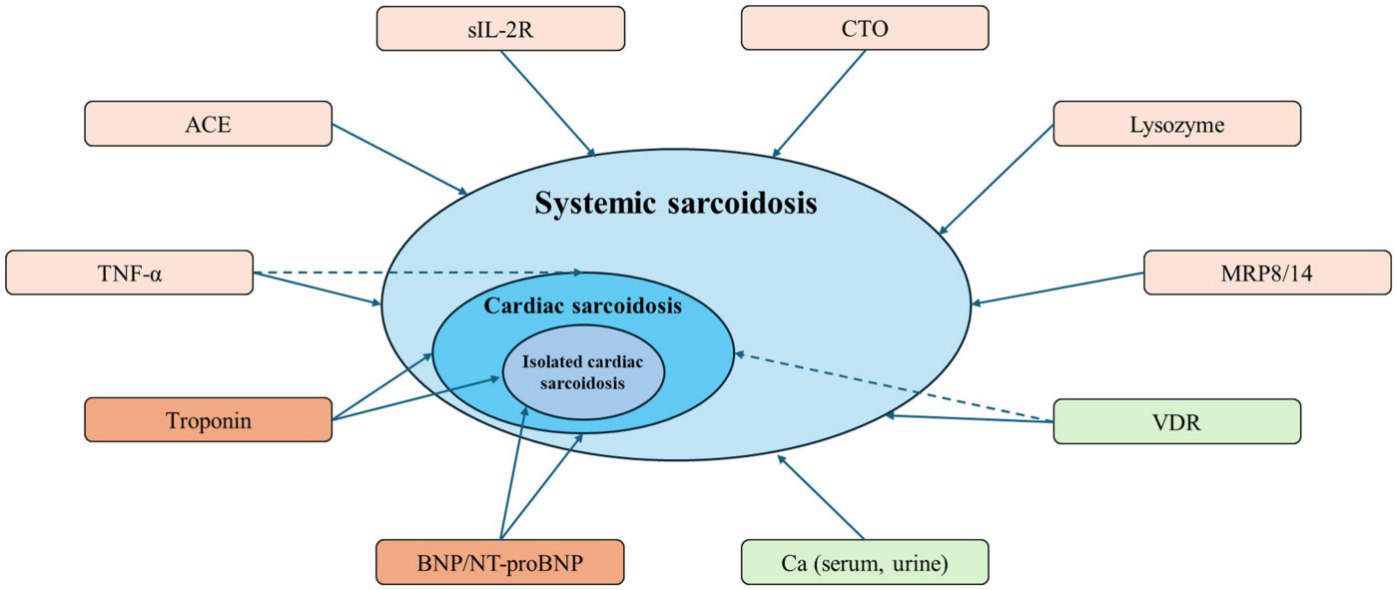
Sarcoidosis is shown as a spectrum from systemic sarcoidosis to cardiac sarcoidosis (CS) and isolated CS. Peripheral blood and metabolic biomarkers that have been studied in this context are placed around the phenotype schema. Solid arrows indicate biomarkers with reported clinical association and potential diagnostic or risk-stratification utility for cardiac involvement. Dashed arrows denote factors with a proposed pathophysiologic relationship to cardiac involvement rather than a validated diagnostic. ACE, angiotensin-converting enzyme; sIL-2R, soluble interleukin-2 receptor; CTO, chitotriosidase; MRP8/14, myeloid-related protein 8/14; TNF-α, tumor necrosis factor alpha; VDR, Vitamin D ratio; BNP, B-type natriuretic peptide; NT-proBNP, N-terminal pro–B-type natriuretic peptide; Ca, Calcium.

In sarcoidosis, serum ACE, sIL-2R, lysozyme, tumor necrosis factor alpha (TNF-α), myeloid-related protein complex (MRP8/14) and chitotriosidase (CTO) (Table 1; Figure 1) reflect monocyte-macrophage activity within granulomas (8, 9). Their performance in isolated CS, however, is constrained by the relatively low myocardial granuloma burden compared with systemic disease, limiting sensitivity.

**Table 1:** Summary of serum biomarkers discussed in relation to cardiac sarcoidosis, outlining their reported relevance and potential avenues for improvement or future research.

Biomarker	Performance (Study/AUC/sensitivity/specificity/ cut-off/ CS diagnostic criteria)	Relevance in CS	Potential improvements and future directions
	Markers of granulomatous inflammation		
ACE (10–12)	[10]/NR/6.7%/NR/>70 U/L/HRS [11]/NR/24.4%/NR/NR/EMB or extracardiac histology + compatible cardiac findings [12]/0.63/NR/NR/NR/ Multimodal assessment ± biopsy	Very low sensitivity across CS phenotypes; elevated in only a minority of patients with isolated CS; strongly influenced by ACE I/D genotype, comorbidities, and medications.	Genotype-adjusted and context-specific cut-offs; incorporation into multiparametric biomarker panels.
sIL-2R (12)	[12]/0.64/NR/NR/NR/ Multimodal assessment ± biopsy	Low sensitivity; non–cardiac-specific; limited evidence in CS.	Integration into composite biomarker scores rather than stand-alone testing.
Lysozyme (11)	[11]/NR/54.1%/NR/NR/EMB or extracardiac histology + compatible cardiac findings	Low sensitivity; non–cardiac-specific; limited evidence in CS.	Integration into composite biomarker scores rather than stand-alone testing.
CTO (32)	[32]/NR/100%/NR/>45 nmol/h/mL/ Extracardiac histology + imaging	More frequently elevated than ACE in systemic sarcoidosis with cardiac involvement; strongly genotype-influenced; non–cardiac-specific; limited CS-specific data.	Genotype-adjusted and context-specific cut-offs; incorporation into multiparametric biomarker panels.
MRP8/14 (29)	[29]/NR/NR/NR/NR/EMB	May differentiate CS from systemic sarcoidosis without cardiac involvement and from dilated cardiomyopathy; evidence limited by small sample sizes.	Validation in larger, phenotype-stratified cohorts; dedicated assessment in isolated CS.
TNF-α (circulating levels) (26)	[26]/NR/NR/NR/NR/ Imaging/biopsy	No association with cardiac involvement in a small, heavily immunosuppressed cohort; diagnostic utility in CS remains uncertain.	Adequately powered, treatment-stratified studies to determine whether TNF-α aids risk stratification of cardiac involvement in pulmonary sarcoidosis.
	Markers of calcium dysregulation and vitamin D metabolism		
Calcium (serum, urine) (11)	Calcium urine: [11]/NR/48.9%/NR/NR/EMB or extracardiac histology + compatible cardiac findings	Hypercalciuria more common in CS with extracardiac disease than in clinically isolated CS; serum calcium does not differentiate cardiac from non-cardiac sarcoidosis.	Integration into composite biomarker scores rather than stand-alone testing.
VDR (37)	[37]/NR/NR/NR/NR/HRS	Low VDR was independently predictive of cardiac involvement.	Prospective validation; phenotype-specific cut-offs; integration with inflammatory and cardiac biomarkers.
	Markers of cardiac injury and distress		
BNP/NT-proBNP (12, 40, 41)	BNP: [12]/0.85/85.4%/68.1%/40 pg/mL/ Multimodal assessment ± cardiac/extracardiac biopsy [40]/0.67/52.4%/91.3%/20 pg/mL/JCS NT-proBNP: [41]/0.913/86.2%/90.1%/213 pg/mL/JCS	Higher in CS than in extracardiac sarcoidosis; good diagnostic accuracy at optimized cut-offs.	Employ as rule-in or risk-stratification markers within multiparametric algorithms, rather than as stand-alone diagnostic tests.
Troponin I/hs-cTnT (12, 42, 43)	Troponin I: [12]/0.58/NR/NR/NR/ Multimodal assessment ± biopsy hs-cTnT: [42]/NR/87.5%/75.0%/>0.014 ng/mL/JCS [43]-reporting prognostic performance	Often elevated and may detect subtle myocardial injury; limited discriminatory ability between CS and non-cardiac sarcoidosis in mixed cohorts.	Employ as rule-in or risk-stratification markers within multiparametric algorithms, rather than as stand-alone diagnostic tests.

CS, cardiac sarcoidosis; ACE, angiotensin-converting enzyme; I/D, insertion/deletion; sIL-2R, soluble interleukin-2 receptor; CTO, chitotriosidase; MRP8/14, myeloid-related protein 8/14; TNF-α, tumor necrosis factor alpha; VDR, Vitamin D ratio; BNP, B-type natriuretic peptide; NT-proBNP, N-terminal pro–B-type natriuretic peptide; hs-cTnT, high-sensitivity cardiac troponin T; NR, Not reported; EMB, Endomyocardial biopsy.

In a cohort of 45 patients with biopsy-proven CS (39 isolated CS; 6 with extracardiac involvement), elevated ACE was observed in only three patients, two of whom had significant pulmonary involvement (10). Similarly, in a nationwide Finnish series of 110 patients with histologically confirmed CS, ACE was elevated in 15% of isolated CS vs. 42% in those with extracardiac involvement (11).

Lysozyme was elevated more often than ACE in isolated CS, but sensitivity remained below 50% (11). Importantly, both biomarkers lacked sufficient sensitivity to diagnose isolated CS, even when patients with extracardiac FDG uptake but no clinical signs of extracardiac sarcoidosis were still classified as having isolated CS. In phenotype-based comparisons, ACE and sIL-2R concentrations were significantly lower in isolated CS than in systemic sarcoidosis with cardiac involvement, supporting limited discriminatory value for detecting isolated myocardial disease (12).

Threshold selection and host factors further complicate interpretation. In ROC analysis, Kawai et al. reported an AUC of 0.865 for ACE and showed that lowering the cut-off increased sensitivity for sarcoidosis (42% vs. 78%) with an expected reduction in specificity (99% vs. 82%) (13). Although conducted in a mixed sarcoidosis population, ACE concentrations did not differ significantly across systemic sarcoidosis, systemic sarcoidosis with cardiac involvement, and isolated CS, suggesting that cut-off optimization could improve case-finding in CS but it is unlikely to resolve specificity limitations. Importantly, generalizability is limited by the exclusively Japanese derivation cohort, and ACE interpretation is further confounded by strong genetic (ACE I/D polymorphism) and clinical modifiers (comorbidities and ACE-inhibitor therapy), arguing against universal thresholds and supporting context-specific calibration (13–15).

TNF-α is central to macrophage activation and granuloma formation, but its diagnostic utility is limited by poor specificity because elevations occur in various acute and chronic inflammatory states (e.g., sepsis, chronic heart failure, rheumatologic disease) (16–18). Nevertheless, in pulmonary sarcoidosis, higher TNF-α levels have been associated with disease progression and extrapulmonary involvement, supporting a role for TNF-α signalling in phenotypic heterogeneity (19–21). Genetic variants linked to increased TNF-α production have also been associated with cardiac involvement, suggesting that TNF-α-related pathways may influence susceptibility to CS, including its isolated forms (22–25).

In contrast, an analysis of circulating cytokines in 98 sarcoidosis patients of African American and European descent found no association between TNF-α levels and cardiac involvement, arguing against TNF-α as a diagnostic marker for CS in that dataset (26). Interpretation is limited by low prevalence of cardiac involvement (11.2%) and substantial treatment confounding: most participants were receiving corticosteroids or had undocumented corticosteroid exposure, many were treated with disease-modifying antirheumatic drugs, and a subset received anti–TNF-α therapy. Together with limited event counts, these factors reduce power and may obscure associations. Rather than supporting standalone diagnostic use, TNF-α may be more appropriately evaluated as part of multimarker or risk-stratification models in clinically relevant, treatment-stratified cohorts.

MRP8/14 is another inflammatory candidate biomarker. This calcium-binding heterodimer is released by activated neutrophils and monocyte/macrophages, including granuloma-associated cells, and has pro-inflammatory and antimicrobial functions (27). MRP14 levels in bronchoalveolar lavage fluid are increased in pulmonary sarcoidosis and idiopathic pulmonary fibrosis compared with healthy controls (28). In serum, patients with systemic sarcoidosis had higher MRP8/14 concentrations than healthy controls (515 vs. 230 ng/mL, *P* = 0.0019) and showed a trend towards higher levels than patients with dilated cardiomyopathy (515 vs. 252 ng/mL, *P* = 0.1224) (29).

Notably, patients with histologically confirmed CS exhibited even higher concentrations (974 ng/mL), exceeding levels in systemic sarcoidosis without cardiac involvement (*P* = 0.0227) and in dilated cardiomyopathy (*P* = 0.0026) (29). These results suggest potential discriminatory value for CS beyond conventional granulomatous inflammation markers. However, the study was constrained by small sample size, non-matched comparison groups, and a CS cohort enriched for advanced disease (all with reduced left ventricular ejection fraction), limiting generalizability.

CTO, a marker of macrophage activity, is an established serum indicator of granulomatous inflammation in systemic sarcoidosis and has been associated with disease progression and specific phenotypes (30, 31). Evidence in CS is sparse. In a small retrospective series of 6 patients with CS and extracardiac involvement, CTO was markedly elevated in all cases despite normal ACE concentrations, suggesting potential incremental sensitivity over ACE in this setting (32). These findings suggest that CTO may provide incremental diagnostic value in CS, with greater sensitivity than ACE. Interpretation is tempered by the strong genetic influence on CTO: relatively common polymorphisms affect both enzymatic activity and circulating levels, complicating threshold-based diagnostic use without genotype context (33).

More broadly, these “granulomatous inflammation” markers (CTO, MRP8/14, ACE, sIL-2R, TNF-α, lysozyme) are limited by imperfect specificity because granulomas also occur in other diseases (e.g., tuberculosis, silicosis), and several markers may be elevated even without granuloma formation (e.g., lysosomal storage disorders, hematologic malignancies) (15, 17, 18, 27, 34). Comparative, phenotype-anchored studies across granulomatous and non-granulomatous mimics could further refine context-specific interpretation and improve diagnostic performance in both systemic sarcoidosis and CS.

## Markers of calcium dysregulation and vitamin D metabolism

Several additional serum indices have been explored for CS, including markers of calcium dysregulation and vitamin D metabolism. In sarcoidosis, 1α-hydroxylase within granulomas can generate 1,25(OH)2D3 (calcitriol) outside normal renal feedback control, predisposing to hypercalcemia and hypercalciuria (35, 36). Being granuloma-dependent, these measures are expected to share a key limitation of other granulomatous inflammation markers – reduced sensitivity when disease is isolated to the myocardium.

Consistent with this, in the Finnish cohort, abnormal urinary calcium was more prevalent in CS with established extracardiac disease than in clinically isolated CS (62% vs. 38%) (11). In a Greek cohort (*n* = 78), serum calcium did not differ by cardiac involvement, but individuals with CS exhibited higher 25(OH)D3 (calcifediol) and a lower 1,25(OH)2D3/25(OH)D3 ratio (VDR) (37). Notably, a low VDR independently predicted cardiac involvement, outperforming even left ventricular ejection fraction. The association persisted after matching for pulmonary disease extent (Scadding stage), suggesting it was not merely a surrogate for radiographic pulmonary disease severity. These data align with the immunomodulatory role of vitamin D and raise the possibility that a low VDR captures a host inflammatory milieu associated with cardiac involvement rather than merely reflecting granuloma mass (38). Moreover, a pure “granuloma burden” explanation would be expected to increase, rather than decrease, the VDR via enhanced 1α-hydroxylase activity, further supporting a phenotype-linked signal.

## Integration of serum biomarkers of granulomatous inflammation

Individual granulomatous inflammation markers are often insensitive in clinically isolated CS, consistent with limited myocardial granuloma burden. A rational mitigation strategy is multimarker integration, in which complementary markers are combined to improve discrimination. Enyedi et al. evaluated ACE, lysozyme, sIL-2R, and CTO for distinguishing sarcoidosis from important mimics, including lymphoma, histiocytosis, and carcinoma) (39). They reported improved diagnostic performance when combining CTO (higher sensitivity) and ACE (higher specificity) as a multiplicative “double product”, increasing AUC from 0.823 (ACE) and 0.861 (CTO) to 0.900.

This framework is particularly relevant for organ-specific phenotyping: integrating markers of granulomatous inflammation with measures of target-organ injury may facilitate earlier recognition of involved organs. For CS, panels coupling inflammatory markers with cardiomyocyte injury indices may better identify patients who merit targeted cardiac evaluation, especially when isolated disease is suspected.

## Markers of cardiac dysfunction and injury

Serum biomarkers of granulomatous inflammation may support sarcoidosis activity but are poorly suited to localizing cardiac involvement in patients with systemic disease. In this setting, conventional cardiac biomarkers (natriuretic peptides and troponins), can provide complementary evidence of myocardial stress and injury and help triage patients for targeted cardiac involvement.

Yasutake et al. compared 27 patients with CS (diagnosed using the criteria of the Specific Diffuse Pulmonary Disease Research Group, Sarcoidosis Division of the Japanese Ministry of Health, Labour and Welfare) with 35 patients with extracardiac sarcoidosis and found higher plasma NT-proBNP in the CS group (40). In contrast, atrial natriuretic peptide (ANP) concentrations were higher in the CS group but did not reach statistical significance. Using a 20 pg/mL cut-off, BNP and ANP showed limited discrimination for cardiac involvement [BNP: 0.67 (0.52–0.81); ANP: 0.61 (0.45–0.77)]. All patients in the study exhibited plasma cardiac troponin T levels <0.01 ng/mL.

Similarly, Kiko et al. observed higher BNP levels in CS and reported improved performance when using a higher threshold [BNP cut-off to 40 pg/mL; AUC: 0.85 (0.75–0.94)] (12). Likewise, NT-proBNP was higher in CS (*n* = 29) than in systemic sarcoidosis without cardiac involvement (*n* = 101)), as per the revised diagnostic criteria of the Japanese Ministry of Health and Welfare (41). At a cut-off value of 213 pg/mL, NT-proBNP demonstrated high diagnostic accuracy (AUC 0.91). Notably, NT-proBNP in CS exceeded levels observed in sarcoidosis with pulmonary hypertension, suggesting that neurohumoral activation may still flag even in advanced pulmonary disease.

Compared to conventional troponin assays, high-sensitivity cardiac troponin (hs-cTnT) assays may detect low-grade myocardial injury often present in CS. In one cohort of systemic sarcoidosis with cardiac involvement (*n* = 12), hs-cTnT was elevated in 8 patients; 3 of the 4 patients with normal hs-cTnT levels had received steroid therapy for ≥ 2 years, consistent with treatment-related attenuation of injury signals (42). In a larger analysis, Baba et al. divided patients into two groups based on hs-cTnT levels. As expected, sarcoidosis patients with elevated hs-cTnT had a higher prevalence of cardiac involvement compared to those with normal hs-cTnT (60% vs. 33%; *P* < 0.001); however, hs-cTnT performed poorly in distinguishing patients with CS from those without (43). This limited discrimination likely reflects treatment and outcome misclassification. Inclusion of patients receiving corticosteroids may attenuate inflammation-related cardiomyocyte injury and lower hs-cTnT. In addition, defining cardiac involvement using relatively insensitive criteria (symptoms, ECG, or echocardiography) may miss subclinical disease, consistent with three patients with elevated hs-cTnT at enrollment who were diagnosed with CS only during follow-up.

Overall, natriuretic peptides and hs-cTnT are best viewed as adjunctive triage tools, raising suspicion and prompting definitive evaluation (CMR/FDG-PET), rather than confirming CS. Their specificity is intrinsically limited because levels are influenced by common conditions (e.g., acute coronary syndromes, heart failure and chronic kidney disease), and interpretation requires clinical context.

## Emerging approaches: omics-derived signatures and ML-enabled triage

Omics profiling may identify circulating molecular signatures that complement CS diagnostic pathways and support non-invasive triage (44–46). Circulating miRNA-126 and miRNA-223 were reported to be higher in CS than in controls and similar in isolated vs. extracardiac CS, suggesting a cardiac-linked signal (47). Although these miRNAs are implicated in immune regulation, the study enrolled only CS patients with heart failure, and heart failure–related neurohumoral activation may have influenced circulating miRNA levels independent of CS (47–49). By contrast, Crouser et al. identified a different miRNA signature, underscoring limited standardization and inconsistent signals to date (50). Collectively current omics signals are best viewed as hypothesis-generating biomarkers that require standardized phenotyping and reproducible assay pipelines before clinical translation.

Machine learning can integrate multivariable laboratory and clinical data to stratify CS risk and guide escalation to definitive testing (CMR/FDG-PET and rhythm evaluation), consistent with broader experience across clinical diagnostic modeling (51, 52). The main barrier is training data: CS is rare, and models derived from homogeneous cohorts may not generalize. International, multi-center datasets with harmonized definitions and rigorous external validation are therefore essential.

## Genetics and cardiac sarcoidosis

Despite more than a century of investigation, sarcoidosis remains etiologically unresolved (53). Current models implicate a gene-environment interaction in which host susceptibility to as-yet unidentified antigens drives a Th1-dominant response and a cytokine milieu enriched in granuloma-promoting mediators, including interferon-gamma (IFN-γ) and interleukin (IL)-2 (54). Genome-wide association studies (GWAS) support a genetic component and repeatedly implicate immune-regulatory loci involved in immune cell differentiation, antigen presentation, and cytokine secretion (54, 55). Although familial clustering, twin concordance, and ethnic/geographic variability point to genetic contribution, the genetic determinants of cardiac involvement remain less clearly defined.

Available observations nonetheless suggest that genetic background may influence the propensity for CS. Early autopsy series reported marked interethnic differences in cardiac involvement among patients with systemic sarcoidosis, with higher rates in Japanese patients than in Caucasian and African American patients (68% vs. 14% vs. 21%) (56). Across clinical cohorts, reported prevalence of cardiac involvement varies widely (3.7%–54.9%), but comparisons are complicated by heterogeneous diagnostic standards (symptom-based criteria vs. advanced imaging vs. histologic confirmation), which can inflate apparent between-study and between-population differences (57).

A familial signal is further supported by a report of monozygotic twin brothers who both developed CS with similar evolution from pulmonary disease to heart failure (58). Genetic testing may thus hold promise for predicting cardiac involvement and disease progression, potentially guiding clinicians in diagnostic and treatment decisions. Nonetheless, the current genetic evidence base remains limited, and most reported associations require confirmation in larger, ethnically diverse cohorts using standardized definitions of cardiac involvement.

Table 2 provides an overview of all genetic variants examined in the context of cardiac sarcoidosis, together with their reported associations and key directions for future research.

**Table 2:** Genetic variants examined in the context of cardiac sarcoidosis, highlighting their proposed relevance and key areas requiring further validation.

Genetic group	Relevance in CS	Potential improvements and future directions
HLA class II variants (61, 62)	Associated with increased susceptibility to CS in Japanese cohorts; relevance in other populations remains uncertain.	Validation in ethnically diverse populations; integration with detailed cardiac and extracardiac phenotyping.
HLA class I variants (62)	Reported risk and protective associations for CS in Japanese cohorts; generalizability to other populations unclear.	Validation in ethnically diverse populations; integration with detailed cardiac and extracardiac phenotyping.
HLA class II heterodimers (62)	Certain DQ/DP haplotypes show stronger CS associations in Japanese cohort and may relate to impaired LVEF; generalizability to other populations unclear.	Validation in ethnically diverse populations; integration with detailed cardiac and extracardiac phenotyping.
TNF-α promoter variants (23–25)	Promoter-enhancing variants more prevalent in CS in Japanese and Greek cohorts; unclear whether they confer sarcoidosis risk overall or are specific for cardiac involvement.	Validation in ethnically diverse populations; direct comparison with non-cardiac sarcoidosis.

CS, cardiac sarcoidosis; HLA, human leukocyte antigen; LVEF, left ventricular ejection fraction; TNF-α, tumor necrosis factor alpha.

## Human leukocyte antigen variants and CS

Human leukocyte antigens (HLA) class I and II shape early adaptive immunity by presenting antigens to T lymphocytes and thereby influencing downstream cytokine programmes and immune-cell recruitment. Because HLA molecules determine peptide-binding specificity, they shape which antigens are presented to T cells and thus influence immune activation. Accordingly, HLA haplotypes are repeatedly associated with immune-mediated diseases, including sarcoidosis, where specific variants may confer susceptibility or protection and may also modify phenotype, including cardiac involvement (59, 60).

In Japanese cohorts, several HLA class II alleles have been enriched in CS. In 26 Japanese patients with CS vs. 247 healthy controls, HLA-DQB106:01 and HLA-DQA101:03 were more frequent, with DQB106:01 homozygosity also enriched (61). Several alleles (HLA-DQB105:01, HLA-DQA101:01, HLA-DRB101:01, and HLA-B07) were absent among CS cases in this series, consistent with possible protection. Yamamoto et al. similarly observed higher frequencies of HLA-DQB106:01, HLA-DQA101:03, and HLA-DRB108:03 in 60 Japanese CS patients than in healthy controls (62). Additionally, HLA class I analysis suggested HLA-A11:01 as a risk allele and HLA-C03:04 as potentially protective.

For class II, analysis at the heterodimer level is biologically coherent because the peptide-binding groove is formed by paired *α* and *β* chains. In Yamamoto et al., several heterodimers were enriched in CS vs. controls, including DQA103:03/DQB106:01, DQA101:03/DQB104:01, and DPA102:02/DPB109:01 (62).

Beyond susceptibility, evidence linking HLA to CS severity or specific clinical phenotypes, such as ventricular tachycardia/fibrillation (VT/VF), complete atrioventricular block (CAVB), sudden cardiac death (SCD) or heart failure (HF), remains limited. Using an HLA-peptide affinity approach against cardiac epitope libraries, Yamamoto et al. reported that HLA-DQA105:0X/DQB103:01 heterodimers were associated with LVEF < 50% at enrolment (90% of CS with the variant vs. 55% without; *P* = 0.043). Notably, whole-exome sequencing did not identify pathogenic cardiomyopathy variants in the HLA-DQA105:0X/DQB103:01 group, whereas such variants were present in the comparison group, supporting, but not proving, an HLA-linked mechanism rather than occult inherited cardiomyopathy (62).

Current HLA associations in CS should be interpreted cautiously. Most studies derive from Japanese cohorts, and studies in Caucasian populations have not replicated these findings (63). This discrepancy may reflect under-recognition of CS in non-Japanese populations, as illustrated by the ACCESS study, in which only 2% of 736 American sarcoidosis patients exhibited cardiac involvement (64).

Furthermore, several studies lack granular data on extracardiac disease, limiting inference on whether alleles are specific to cardiac involvement or track broader sarcoidosis phenotypes (e.g., uveitis) (64).

A more definitive understanding will require ethnically diverse cohorts with standardized CS definitions, systematic characterization of extracardiac involvement, and external validation across centers (65). To address progression, studies should incorporate longitudinal phenotyping, leveraging prior ECG/echocardiography and prospective follow-up, to relate HLA variation to incident cardiac involvement and clinical trajectories. Finally, because HLA effects may be exposure-dependent, comparative designs in similar ancestry groups across different geographic environments could help disentangle genetic susceptibility from environmental triggers (65).

## Tumor necrosis factor-α gene polymorphisms and CS

TNF-α is central to granuloma formation and persistence, and TNF-α inhibition is used in selected refractory sarcoidosis phenotypes, motivating interest in TNF-α promoter variants that influence its expression (22). In Japanese cohorts, promoter alleles associated with higher TNF-α production have been reported more frequently in CS than in controls. TNF-α-308A was enriched in 26 Japanese patients with CS compared with 125 healthy controls (19.2% vs. 1.6%, corrected *P* = 0.003496), and TNF-α-857 T was also more prevalent in 50 Japanese CS patients than in a mixed control group (TT genotype 10% vs. 2.4%, *P* = 0.006) (23, 24). However, these comparisons were made against non-sarcoidosis controls rather than sarcoidosis patients without cardiac involvement, and neither study reported extracardiac disease extent, limiting inference about CS-specific risk (23, 24).

Across other populations (including German, British, Dutch, Czech, Polish, Serbian, Indian, and African American cohorts), TNF-α promoter polymorphisms have shown inconsistent associations with sarcoidosis susceptibility and with Löfgren syndrome (66–75). Because these studies did not evaluate cardiac involvement, their implications for CS remain uncertain. In a study designed to address phenotype, Gialafos et al. compared patients with pulmonary sarcoidosis (*n* = 131) to those with additional cardiac involvement (*n* = 42), with groups balanced for sarcoidosis stage and cardiac involvement defined using modified Japanese criteria (25). The CS group showed higher frequencies of TNF-α-857T and TNF-α-308A (both *p* = 0.0012), extending prior Japanese signals to a Greek population. Whether these variants consistently predict cardiac involvement across ancestries, and whether effects are independent of extracardiac involvement, remains unresolved.

## Other genetic variants associated with CS

Beyond HLA and TNF-α, most candidate genetic signals in sarcoidosis map to immune-regulatory pathways. Butyrophilin-like 2 (BTNL2), a transmembrane immune checkpoint-like molecule that can transmit inhibitory signals to T cells, has been repeatedly linked to systemic sarcoidosis susceptibility in meta-analysis, although CS-specific evidence remains limited (76–79). In a case report of endomyocardial biopsy-proven isolated CS genotyping revealed homozygosity for a BTNL2 splice-altering variant predicted to disrupt the transmembrane anchoring domain and abrogate inhibitory function (80). While biologically plausible, this variant is not CS-specific and has been reported in other sarcoidosis phenotypes and in dilated cardiomyopathy, limiting diagnostic or prognostic inference without larger phenotype-anchored cohorts (81, 82).

Family-based analyses provide another perspective on genetic contribution. In 344 African American affected relative individual-level concordance for cardiac involvement was not observed; however, organ-pattern clustering identified strong linkage (LOD 6.65) at 18q22 for a combined cardiac/renal cluster (83). Within this region, immunoregulatory candidates proposed include suppressor of cytokine signalling 6 (SOCS6) and CD226, genes implicated in multiple autoimmune diseases, although their roles in sarcoidosis and CS remain to be established (84, 85). Finally, in a European cohort, a SNP near LOC102723568 and a SNP in Toll-like receptor 3 (TLR3) were associated with an ocular–cardio–cutaneous–CNS phenotype in a Serbian sub-cohort (86). However, because cardiac involvement was embedded within a composite phenotype, relevance to isolated CS cannot be inferred.

## The overlap of cardiac sarcoidosis and genetic cardiomyopathies

Improved availability of CMR and PET-CT has increased recognition of clinically isolated CS, reported to account for approximately 25% of CS cases (87). However, key imaging hallmarks used to support CS, late gadolinium enhancement and fluorodeoxyglucose uptake, are not disease-specific and can overlap with genetic cardiomyopathies and other inflammatory or infiltrative processes.

In a retrospective Mayo Clinic series of 47 patients evaluated for suspected CS (43 isolated; 4 with extracardiac sarcoidosis), genetic testing identified pathogenic/likely pathogenic (P/LP) variants with established or probable cardiomyopathy causality in 10 patients (21%) (88). All variant-positive patients exhibited both late gadolinium enhancement and fluorodeoxyglucose uptake, yet among those who underwent endomyocardial biopsy (*n* = 7), none had histologic granulomatous inflammation, highlighting the limited specificity of imaging-based CS attribution in this context. Notably, 1 variant-positive patient also had systemic sarcoidosis, indicating that genetic testing can remain informative even when sarcoidosis is present extracardially.

Additional complexity arises, as cases have been reported in which biopsy-proven CS coexists with P/LP variants associated with genetic cardiomyopathies (89). Compared to patients with genetic cardiomyopathy alone, those with both CS and P/LP variants exhibited significantly higher rates of atrial and atrioventricular conduction abnormalities, elevated NT-proBNP and CRP levels, and greater septal involvement on imaging. Although this study included only 10 patients, the groups were matched for demographic characteristics and P/LP variants, suggesting that CS may account for these observed differences.

Collectively, these data support incorporating cardiomyopathy gene testing into the evaluation of suspected CS, particularly when disease appears isolated, because identification of a genetic substrate can significantly alter management and family screening. At the same time, detection of a P/LP variant should not be used to “rule out” CS, given documented overlap. Mechanistically, one plausible model is that genetically mediated cardiomyocyte injury may increase cardiac antigen release and local danger signalling, potentially lowering the threshold for granulomatous inflammation in immunogenetically susceptible hosts; this remains hypothetical and requires dedicated genotype–phenotype studies with standardized CS adjudication.

## Future prospects

Epigenetic profiling captures regulatory variation beyond DNA sequence and may reflect gene–environment interactions relevant to sarcoidosis, with potential utility for risk stratification and as modifiable therapeutic targets (47, 90, 91). In pulmonary sarcoidosis, genome-wide methylation profiling of bronchoalveolar lavage cells showed differential methylation enriched in immune/inflammatory regulatory regions; while single loci were not strongly linked to progression, composite signatures predicted progressive disease (90). These observations motivate CS-focused studies assessing whether cardiac-relevant epigenetic signatures can identify isolated CS and stratify cardiac involvement risk in systemic sarcoidosis.

## Conclusion

Circulating biomarkers offer a practical, non-invasive complement to imaging for cardiac sarcoidosis, but single inflammatory markers remain limited, particularly in isolated cardiac involvement, because they lack adequate sensitivity and do not reliably indicate myocardial disease. Diagnostic performance is most likely to improve through standardized cut-off values, multimarker approaches that combine systemic inflammatory signals with cardiac injury/stress biomarkers, and validation in well-phenotyped cohorts with standardized endpoints and sampling timepoints.

Genetic analysis, especially HLA haplotypes and other susceptibility variants, are promising for identifying individuals at higher risk of cardiac involvement and for refining sarcoidosis phenotypes, yet remain underpowered and inconsistently replicated. Integrating biomarkers and genotype with imaging and clinical features, supported by modern molecular profiling and AI-based models, represents the most plausible path toward clinically implementable, personalized risk stratification and longitudinal disease monitoring.
